# Case report: EGFR-TKI rechallenge after osimertinib-induced interstitial lung disease: a case report and literature review

**DOI:** 10.3389/fphar.2024.1410684

**Published:** 2024-06-04

**Authors:** Xiaofei Gu, Yonghong Zhong, Huaqiong Huang

**Affiliations:** ^1^ Department of Respiratory and Critical Care Medicine, Linping Campus, The Second Affiliated Hospital of Zhejiang University School of Medicine, Hangzhou, Zhejiang, China; ^2^ Department of Respiratory and Critical Care Medicine, The Second Affiliated Hospital of Zhejiang University School of Medicine, Hangzhou, Zhejiang, China

**Keywords:** epidermal growth factor receptor-tyrosine kinase inhibitor (EGFR-TKI), rechallenge, osimertinib, non-small cell lung cancer (NSCLC), interstitial lung disease (ILD)

## Abstract

**Background:**

Osimertinib, a third-generation tyrosine kinase inhibitor (TKI), has demonstrated significant efficacy in treating non-small cell lung cancer (NSCLC) patients with epidermal growth factor receptor (EGFR) mutations. However, EGFR-TKI-induced interstitial lung disease (ILD), a well-known adverse effect, can seriously affect the treatment outcome. There is currently no international consensus on the efficacy and safety of re-administration of EGFR-TKI after EGFR-TKI-induced ILD.

**Case summary:**

We report a case of a 62-year-old male with stage IV lung adenocarcinoma and EGFR L858R mutation who was treated with osimertinib at a dose of 80 mg/day as first-line therapy. On the sixth day of treatment, the patient developed grade 4 ILD, chest tightness, shortness of breath, and paroxysmal dry cough. Arterial blood gas analysis indicated the presence of type I respiratory failure, while the chest CT scan revealed newly developed ground-glass opacities in both lungs and a considerable amount of pleural effusion on the left side. Subsequently, the patient was administered methylprednisolone for anti-inflammatory therapy, in conjunction with oxygen therapy, anti-infection treatment, and closed thoracic drainage, which resulted in a favourable recovery and discharge after 18 days. During this period, the patient adhered to third-generation EGFR-TKI oral targeted therapy. Nevertheless, within a week of discharge, the patient was readmitted due to the recurrence of chest tightness and shortness of breath. A chest CT scan indicated a recurrent ILD. Despite the administration of high-dose methylprednisolone for 9 days, the patient’s condition continued to deteriorate, ultimately resulting in death.

**Conclusion:**

It is of the utmost importance to conduct a meticulous evaluation of the severity of osimertinib-induced ILD in order to ascertain the potential risks and benefits of EGFR-TKI rechallenge. Particularly, for patients with grade 4 ILD, firm drug discontinuation should be considered.

## 1 Introduction

In recent years, epidermal growth factor receptor (EGFR) mutations have attracted widespread attention in the treatment of non-small cell lung cancer (NSCLC). The most prevalent types of EGFR mutations are the exon 19 deletion and the exon 21 L858R mutation, collectively accounting for 85%–90% of cases. These mutations are closely correlated with the development and prognosis of NSCLC. Osimertinib, an oral tyrosine kinase inhibitor (TKI) that targets these mutations, has demonstrated substantial efficacy in the treatment of patients with this specific subset of NSCLC (Li et al., 2022).

Despite the clear efficacy of osimertinib, its use brings along significant risks that cannot be overlooked. A number of studies have indicated that patients with EGFR-mutated NSCLC undergoing osimertinib treatment may be susceptible to developing interstitial lung disease (ILD), which can potentially lead to treatment interruptions, medication adjustments, and even jeopardise the patient’s life safety (Gemma et al., 2020). This report presents a case of a lung adenocarcinoma patient with an EGFR L858R mutation who failed a third-generation EGFR-TKI rechallenge after recovering from initial osimertinib-induced ILD, along with a literature review.

## 2 Case presentation

**FIGURE 1:**
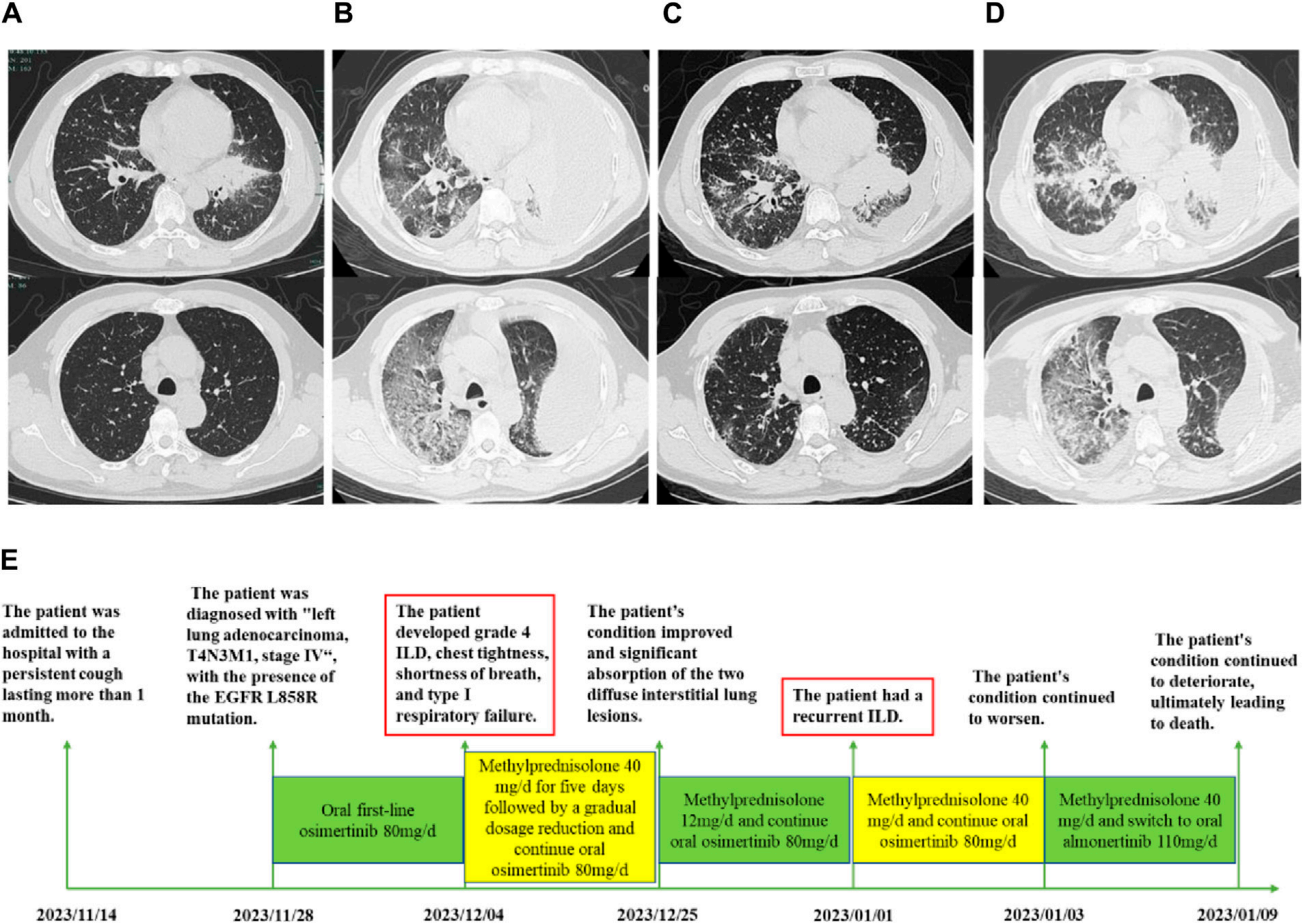
Fi **(A–D)** The clinical course according to CT scan findings. **(A)** The baseline CT scan revealed a mass in the basal segment of the lower lobe of the left lung, with no signs of pre-existing interstitial pneumonia before osimertinib therapy. **(B)** After 6 days of treatment with osimertinib, chest CT showed diffuse ground-glass opacities in both lungs, with the right side being more pronounced and a significant pleural effusion on the left side. **(C)** Following 7 days of methylprednisolone treatment, a repeat chest CT demonstrated significant resorption of the diffuse interstitial lesion identified in the previous scan, indicating a positive response to therapy. **(D)** On 1 January 2024, a follow-up chest CT scan displayed new diffuse ground-glass opacities in both lungs, particularly pronounced in the right lung. **(E)** A thorough timeline of the patient’s clinical progression and treatment protocol.

A 62-year-old male with a history of smoking of over 30 years, consuming approximately 20 cigarettes per day, was admitted to the hospital on 14 November 2023 with a persistent cough that had lasted for over a month. A chest computed tomography (CT) scan revealed bronchial stenosis and occlusion in the lingual segment and dorsal segment of the lower lobe of the left lung, accompanied by peripheral flaky hyperdense shadows, diffuse nodular shadows in both lungs and enlarged lymph nodes in the mediastinum and hilar lymph nodes (Figure 1A). Subsequent laboratory investigations yielded results within the normal ranges for both the blood routine and carcinoembryonic antigen (CEA). It is noteworthy that the glycocalyx antigen 19–9 (CA19-9) was elevated at 160.10 u/mL (normal range <31 u/mL), the cytokeratin 19 fragments (CYFRA21-1) measured 12.72 ng/mL (normal range <4.1 ng/mL), and the neuron-specific enolase (NSE) levels were slightly elevated at 6.46 ng/mL (normal range <6 ng/mL). Bronchoscopy revealed the presence of a neoplasm in the anterior basal segment within the left lower lobe and a nodular protrusion at the lower end of the right main bronchus. Pathological analysis revealed a low-differentiated adenocarcinoma, with immunohistochemistry results indicating the following: P40 (−) TTF-1 (weak+), NapsinA (+) CK7(+) CD56 (−) CK20(−) Syn (−) Ki67(40%). Additionally, cranial magnetic resonance imaging revealed the presence of multiple metastases in both frontal lobes. The positron emission tomography-computed tomography (PET-CT) imaging results revealed the presence of metastatic tumours in the central left lung, with a double neck classification of III-IV. Additionally, there were mediastinal metastases (1R, 2R, 3R, 4R/L, 5/7), double hilar lymph node metastases, multiple intrapulmonary metastases, a left pleural metastasis, and systemic bone metastasis (multiple ribs, vertebrae, and iliac bones on the left side; and femur on the right side). Consequently, the patient was diagnosed with “left lung adenocarcinoma, T4N3M1, stage IV” according to the eighth edition of the American Joint Committee on Cancer (AJCC) staging system. Furthermore, a genetic test indicated the presence of the EGFR L858R mutation, which led to the initiation of osimertinib 80 mg/d orally targeted therapy, commencing on 28 November 2023.

On the sixth day of osimertinib treatment, the patient exhibited signs of respiratory distress, including chest tightness and shortness of breath, accompanied by a paroxysmal dry cough. The arterial blood gas analysis indicated a pH of 7.50, a partial pressure of carbon dioxide (CO_2_) of 30.9 mmHg, a partial pressure of oxygen of 49 mmHg, and an oxygen saturation of 86.8%. The blood routine and ultrasensitive C-reactive protein (CRP) tests demonstrated a leukocyte count of 14.4*10^9/L, a neutrophil percentage of 83.6%, an eosinophil percentage of 0.4%, and a CRP concentration of 143.6 mg/L. However, the rheumatology-related immune indexes, including cANCA, pANCA, PR3/MPOC assay, and antinuclear antibody, were found to be negative. A chest CT scan revealed diffuse ground glass shadows in both lungs, with the right side being more prominent and a large pleural effusion on the left side (Figure 1B). The tumour markers in the pleural and abdominal fluids were CEA 8.96 ng/mL, glycocalyx antigen 125 (CA125) 4958.53 U/mL, and CYFRA21-1 372.38 ng/mL. The presence of cancer cells in the pleural fluid was confirmed through liquid-based thin-layer cytography, which aligns with the suspected diagnosis of adenocarcinoma based on the patient’s medical history. Due to the patient’s weakness and unwillingness, bronchoscopy and alveolar lavage were not performed. The patient did not display any signs of heart failure, was not on amiodarone or immune checkpoint inhibitors, and was not taking any other medications known to induce ILD. In light of the aforementioned considerations, the diagnostic possibilities included new lung infection, lung cancer progression, and EGFR-TKI-associated ILD. Following the administration of methylprednisolone at a dose of 40 mg per day for 5 days, the patient’s condition demonstrated a favourable response, with the addition of oxygen therapy, anti-infective medication, closed chest drainage, and other symptomatic treatments. A follow-up chest CT scan demonstrated significant absorption of the two diffuse interstitial lung lesions in comparison to the previous scan (Figure 1C). By the time of discharge on 25 December 2023, inflammatory markers had returned to normal levels, while the patient continued to receive oral targeted treatment with osimertinib at a dose of 80 mg per day.

On 1 January 2024, 1 week after the initial admission, the patient was readmitted to the hospital due to the onset of symptoms of chest tightness and urgency. Arterial blood gas analysis demonstrated signs of inadequate oxygenation with nasal cannula at 2 L/min, as indicated by a pH level of 7.47, a partial pressure of CO_2_ at 30.5 mmHg, a partial pressure of oxygen at 66 mmHg, and an oxygen saturation level of 94.9. The results of the haematological examination revealed a leukocyte count of 9.1*10^9/L, an eosinophil count of 3.7%, and a CRP level of 31.0 mg/L. Furthermore, tumour markers CEA and CA19-9 were found to be elevated at 5.91 ng/mL and 529.16U/mL, respectively, with NSE levels at 12.40 ng/mL. A chest CT scan revealed the presence of new diffuse ground-glass shadows, predominantly in the right lung (see Figure 1D). To address these potential complications, the patient was administered high-dose methylprednisolone in combination with anti-infective medications, oxygen therapy, and other symptomatic treatments. In light of these developments, it was recommended that osimertinib be discontinued. The patient’s family members independently proceeded to the hospital’s oncology department to commence almonertinib 110 mg/d targeted therapy on 3 January 2024, with an awareness of the heightened risk of disease progression. Despite the aforementioned treatment efforts, the patient’s condition continued to deteriorate, resulting in the onset of progressive respiratory failure, which ultimately led to the patient’s discharge from the hospital on 9 January 2024. A detailed timeline of the patient’s clinical course and therapy regimen is presented in Figure 1E.

## 3 Discussion

Here, we reported a case of a patient who recovered from grade 4 ILD induced by first-line osimertinib treatment but had a recurrent and fatal ILD on subsequent third-generation TKI re-administration therapy.

Osimertinib represents the inaugural third-generation EGFR-TKI to be approved for initial therapy in patients with EGFR-mutated advanced NSCLC. It has been demonstrated to significantly prolong progression-free survival and overall survival in these patients, when compared to first-generation EGFR-TKIs (Lamb, 2021). Nevertheless, osimertinib-induced ILD can be a significant and potentially fatal adverse event that can severely impact treatment outcomes. A clinical study conducted in Japan has revealed that among patients with EGFR mutation-positive NSCLC treated with osimertinib, the incidence of ILD was 6.8%, with a mortality rate of 0.8%, and a median onset time of 63 days (ranging from 5 to 410 days) after treatment initiation (Gemma et al., 2020). Moreover, several risk factors have been identified for ILD in EGFR-TKI-treated patients. These include being male, aged 55 years or older, a history of smoking, less than 50% of normal lung tissue on imaging, a time to definitive cancer diagnosis of less than 6 months, a performance status (PS) score higher than two points, recent radiotherapy, presence of emphysema or chronic obstructive pulmonary disease, a history of interstitial lung disease, a concurrent cardiovascular disease, and a lung infection (Chinese Society of Lung Cancer, 2019).

In this case, the patient exhibited dyspnoea on day 6 following the administration of osimertinib, and a chest CT scan revealed ILD and pleural effusion. Arterial blood gas analysis indicated the presence of type I respiratory failure. It is noteworthy that the patient exhibited several risk factors associated with EGFR-TKI-induced ILD, including being an elderly male with a history of smoking, a definitive diagnosis of lung cancer less than 1 month prior, a PS score of 3, and imaging studies revealing less than 50% normal lung tissue.

The specific mechanism underlying the development of EGFR-TKI-induced Interstitial Lung Disease (ILD) remains elusive. It is speculated to be associated with lung injury, lung fibrosis, and drug-triggered immune responses. EGFR-TKIs are believed to exacerbate lung injury by hindering the regeneration of airway epithelial cells and impeding the repair process. Additionally, they may induce lung fibrosis by stimulating fibroblast migration and proliferation (Chinese Society of Lung Cancer, 2019). Research has shown that heat shock protein-70 (HSP-70) exerts a protective effect against pulmonary fibrosis. However, EGFR-TKIs have been found to suppress HSP-70 expression in the lungs, which may potentially promote pulmonary fibrosis (Enomoto, 2022). Furthermore, the efficacy of EGFR-TKI-induced glucocorticoid treatment in ILD implies a possible link to immune mediation (Kodama et al., 2021).

In accordance with the Common Terminology Criteria for Adverse Events (CTCAE), the administration of EGFR-TKI should be terminated immediately in the event of EGFR-TKI-associated ILD. Nevertheless, there have been documented instances in the literature where successful rechallenge with osimertinib has been achieved following osimertinib-induced ILD. The viable strategies for a successful rechallenge include: 1) Administering an equal or reduced dosage of osimertinib, with or without systemic steroids (Kodama et al., 2021); 2) Switching to EGFR-TKI-targeted therapies other than osimertinib, such as erlotinib, afatinib, gefitinib, and almonertinib (Nishima et al., 2021; Wu et al., 2021; Kanaji et al., 2024). It is notable that the majority of successful rechallenge cases have been reported in patients with ILD grades 1–2. To address this, we believe that it is necessary to further assess ILD severity for differentiation. For instance, our patient was classified as grade 4, with an oxygen saturation of 86.8% and type I respiratory failure. The patient did not discontinue osimertinib but was later replaced with almonertinib, which has been demonstrated to have a lower risk of inducing ILD (Lu et al., 2022). Despite the implementation of aggressive anti-inflammatory and anti-infective interventions, the patient’s condition continued to deteriorate.

This case study illustrates the necessity for a meticulous assessment of the risks and benefits of EGFR-TKI rechallenge in patients with osimertinib-induced ILD, with particular attention to the severity of the ILD. It is of paramount importance to direct particular attention to grade 4 EGFR-TKI-associated ILD, with a clear prioritisation of acute and potentially fatal ILD over the use of aggressive oncologic therapy. In cases of such severity, it is recommended that the TKIs be discontinued as soon as possible. It is recommended that respiratory function be monitored more closely and that imaging examinations be conducted more frequently during treatment in order to enable the early detection, discontinuation, and treatment of any adverse effects. Further research is required to explore sequential therapy strategies for grade 4 osimertinib-induced ILD.

## Data Availability

The original contributions presented in the study are included in the article/Supplementary material, further inquiries can be directed to the corresponding author.
